# Early Feasibility Study to Evaluate the Viveve System for Female Stress Urinary Incontinence: Interim 6-Month Report

**DOI:** 10.1089/jwh.2018.7567

**Published:** 2020-03-17

**Authors:** Bruce B. Allan, Stacie Bell, Kathryn Husarek

**Affiliations:** ^1^ Allan Centre, Calgary, Canada.; ^2^ Viveve, Inc., Englewood, Colorado.

**Keywords:** one-hour pad-weight test (1-hour PWT), radiofrequency (RF), stress urinary incontinence (SUI)

## Abstract

***Background:*** The purpose of this prospective, investigator-initiated feasibility study is to evaluate the efficacy and safety of nonablative, cryogen-cooled, monopolar radiofrequency (CMRF) treatment for stress urinary incontinence (SUI).

***Materials and Methods:*** Subjects meeting all the inclusion and exclusion criteria were enrolled and divided into two groups. Subjects in Group 1 received a single SUI treatment, and subjects in Group 2 received two SUI treatments ∼6 weeks apart. Follow-up visits are planned for 1, 4, 6, and 12 months post-treatment. At each study visit, subjects are asked to perform a 1-hour pad-weight test (PWT) and to complete the Urogenital Distress Inventory-6 (UDI-6), Incontinence Impact Questionnaire-Short Form (IIQ-7), and International Consultation on Incontinence Modular Questionnaire-Urinary Incontinence-Short Form (ICIQ-UI-SF) questionnaires. In addition, subjects completed 7-day bladder voiding diary and safety assessments.

***Results:*** Preliminary data indicate an improvement in SUI symptoms and quality of life for subjects, as determined by validated SUI-related patient-reported outcomes and the objective 1-hour PWT, with a >50% reduction in pad weight for 68.8% of the Group 1 subjects and 69.2% of the Group 2 subjects at 6 months. Initial review of the bladder voiding diaries suggests that subjects are having fewer urine leakage episodes per day. In addition to efficacy, the CMRF Viveve System was well tolerated and safe.

***Conclusions:*** The endpoints evaluated indicate an improvement in SUI symptoms and quality of life. The sustained benefit of the CMRF vaginal treatment at 6 months suggests potential use as a nonsurgical approach to treat SUI.

## Introduction

Urinary incontinence is the involuntary loss of urine. There are two major types of female urinary incontinence: urgency urinary incontinence and stress urinary incontinence (SUI). Urgency incontinence is the complaint of involuntary loss of urine associated with the sudden need to pass urine.^1^ Stress incontinence is the involuntary loss of urine after a cough, sneeze, or physical activity.^1^ SUI is the most prevalent type of UI in women^2^ and has two major subtypes: intrinsic sphincter deficiency (ISD) and urethral hypermobility. Patients with ISD leak urine because the urethral sphincter does not effectively seal off the inner muscle of the bladder. Urethral hypermobility refers to the movement of the female urethra that occurs due to weakened pelvic floor muscles. In reality, many women have a mixed presentation.

SUI affects many women; especially during pregnancy, after childbirth, and during menopause. More than 55% of women who have delivered a child vaginally will show symptoms of SUI and are twice as likely to suffer from long-term SUI when compared with cesarean delivery.^3^ Furthermore, SUI can greatly impact a woman's health and quality of life.^4^ Depending on the severity of incontinence, some women may choose to avoid social or religious gathering, physical exercise, travel, and even sex.^5,6^

There are various treatment possibilities for women suffering from SUI; however, the current options have many limitations. Conservative, first-line treatment options include simple diet and exercise changes, and pelvic floor muscle training. Some women may find benefit from these,^7^ but long-term compliance and sustainability is difficult.^8^ Pharmacologic intervention^9^ or injectable bulking agents^10^ offer additional conservative treatment options but may pose efficacy or safety issues.^11^ At the other end of the treatment spectrum is surgery with mesh or a sling. Although synthetic sling placement has a proven success rate,^12^ complications of mesh surgery can occur, negatively impacting many patients. In addition, surgery often comes with several risks, including infection, voiding dysfunction, and anesthetic concerns,^13^ leading many women to use surgery as a last resort for treatment. The large gap between conservative and highly invasive treatment options presents an opportunity to provide more effective and less-invasive treatments for women suffering from SUI.

Radiofrequency (RF) energy has previously been used in various mucosal tissues, including pharynx, skin, cornea, and vagina.^14,15^ In addition, RF devices have been used to treat a variety of health-related issues, including SUI.^16^ However, these precedent devices are no longer commercially available as there have been some concerns about their safety profile.^17–19^ The Viveve System, a cryogen-cooled monopolar RF device, has a well-documented safety profile and has previously been used to treat sexual dysfunction.^15,20^ The device delivers monopolar RF with cryogen cooling to protect the upper epithelial layers of the mucosa while also enabling energy to reach the deeper tissue layers, resulting in volumetric heating of important connective tissue.

Recently, a pilot study at the Allan Centre in Calgary, Canada using the Viveve System to treat women with SUI reported a >90% improvement from baseline in SUI symptoms and quality of life (data on file). Following those positive results, this larger investigator-initiated early feasibility study is being conducted to further evaluate the use of the Viveve System to treat SUI. This study includes the objective 1-hour pad-weight test (PWT) in addition to subjective patient-reported outcome measures.

## Materials and Methods

### Study design and research subjects

This investigator-initiated feasibility study is a single-site, randomized, unblinded trial that is ongoing.

**FIG. 1.**
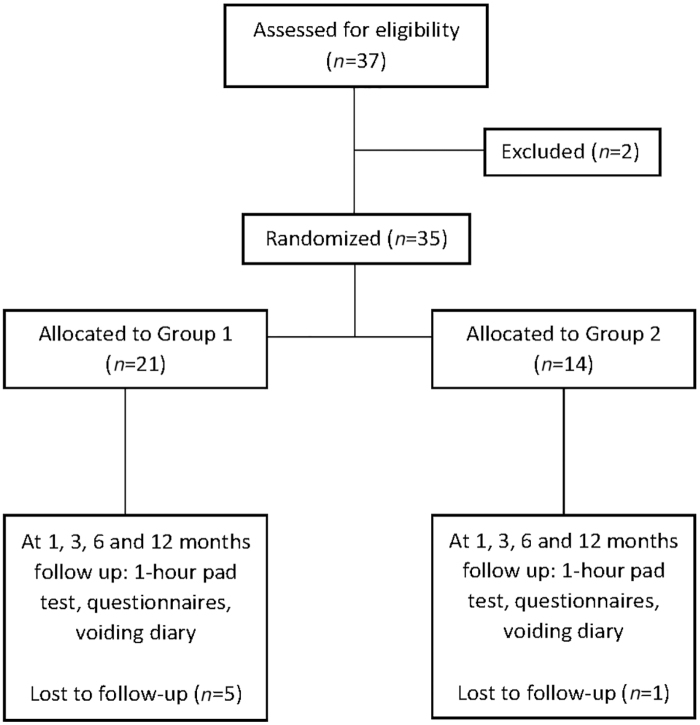
CONSORT diagram. Note, the final follow-up visit is at 12 months, but this article only discusses the trial through 6-month follow-up visit.

The trial included females (≥18 years of age) with a normal pelvic examination who were diagnosed with mild-to-moderate SUI as defined by the 1-hour PWT (1–50 g leakage).^21^ Women were excluded from the trial who were currently pregnant or discontinued breast feeding <6 months before enrollment; had a condition/illness that may confound the results of urinary incontinence assessment, including an abnormal pelvic examination, greater than stage II pelvic organ prolapse, or were morbidly obese; had a history of genital fistula or a thin rectovaginal septum (<2 cm); had a previous energy-based device treatment in the genitourinary area; and/or were taking any new medication that affects urination (Fig. 1).

### Randomization and intervention

Subjects meeting the inclusion and exclusion criteria were randomized to receive either one or two treatments using a random number generator; odd numbers were placed into Group 1 and even numbers were placed into Group 2. The Viveve system protocol for sexual function^15^ was modified to provide additional energy to the tissue beyond the introitus for support of the urethra to improve SUI. A total of 220 pulses of 90 J/cm^2^ was applied during the treatment procedure. The treatment area was divided into quadrants of the vaginal introitus with the area directly beneath the urethra excluded. Each quadrant was treated with five consecutive passes of five locations of pulses for a total of 25 pulses per quadrant. The remaining 20 pulses are distributed equally in quadrants 1 and 4. If a subject was assigned to Group 2 (two SUI treatments), the treatment protocol was repeated 6 weeks after the initial treatment.

A sham group was not included in the study design as this was a pilot study to obtain the first objective data using the Viveve Treatment, SUI protocol. In addition, the manufacture of a sham tip would have been cost prohibitive at this stage in the investigation.

The sample size (37 subjects) for this early feasibility study was determined based on considerations of such a feasibility study, confidence in outcome measures, and research risks, including the risk that the study aims may not be achieved.

### Follow-up visits

The standardized 1-hour PWT,^21^ 7-day bladder voiding diary and validated patient-reported outcome measures (Urogenital Distress Inventory-6 [UDI-6],^22^ Incontinence Impact Questionnaire-Short Form [IIQ-7],^22^ and International Consultation on Incontinence Modular Questionnaire-Urinary Incontinence-Short Form [ICIQ-UI-SF]^23^), was administered at the screening visit and at 1, 4, and 6 months post-treatment. These will also be completed at the final visit at 12 months. Adverse events and concomitant medications were collected at each of the follow-up visits.

### Objective 1-hour PWT

The 1-hour PWT is a standardized series of activities (walking, coughing, climbing stairs, *etc.*) that the subject completes after ingestion of a set amount (500 mL) of sodium-free liquid. Subjects are asked to wear preweighed pads during the assessment. The pad is weighed again at the completion of the series of activities to determine the amount of leakage.

### Subjective patient-reported outcomes

The questionnaires used to evaluate the status of the subject's SUI were UDI-6, IIQ-7, and ICIQ-UI-SF. These questionnaires have been used in several SUI clinical trials and have been validated for this use.^22,23^ Scoring was done per the respective questionnaire guidelines.

### Seven-day bladder voiding diary

A site-developed 7-day bladder voiding diary was provided to subjects for completion. The voiding diary included questions about leakage and daily activities.

### Ethics

Ethics Review Board approval was obtained from the Health Research Ethics Board of Alberta, and the study was done in compliance with Good Clinical Practices and International Conference on Harmonization (ICH) guidelines. Health Canada clearance was also obtained for this investigator-initiated study. Documentation and data management were conducted in a manner that aligns with local ethics review board guidelines.

## Results

### Participants

Between June and November 2017, 37 subjects were included in the study. Twenty-one and 14 subjects were randomized to receive one or two treatments, respectively; 2 subjects dropped out of the study before treatment. Twenty-nine subjects completed the baseline and 6-month follow-up visit. Table 1 shows the baseline characteristics for the remaining 29 subjects. Group 2 was slightly older than Group 1, 46.5 years of age versus 42.9, respectively. Both groups had similar BMI and number of pregnancies. A large majority (96%) of the subjects were of white race, and one subject was Asian.

**Table 1.** Baseline Demographics, Clinical Characteristics, and Maternal History for Trial Subjects

No. of subjects	Group 1		Group 2	
	16		13	
Demographic data	Mean	SD	Mean	SD
Age	42.9	4.0	46.5	9.2
Age categories, years	*n*	%	*n*	%
<35	1	6.3	1	7.7
35–39	4	25.0	2	15.3
40–44	4	25.0	3	23.1
≥45	7	43.7	7	53.9
*Clinical data*	*Mean*	*SD*	*Mean*	*SD*
BMI	25.1	4.6	25.9	4.7
BMI categories	*n*	%	*n*	%
BMI <20	3	18.8	0	0
BMI 20–24	5	31.2	5	38.5
BMI 25–29	5	31.2	6	46.1
BMI ≥30	3	18.8	2	15.4
*Maternal history*	*Mean*	*SD*	*Mean*	*SD*
No. of pregnancies	2.0	1.3	2.3	0.9
No. of full-term deliveries	1.7	1.0	2.3	0.8
No. of vaginal deliveries	1.5	1.1	2.0	1.1
Race	*n*	%	*n*	%
White	15	93.8	13	100
Asian	1	3.5	0	0

### One-hour pad weight

Mean 1-hour pad-weight values are presented in Table 2. Baseline values differ between treatment groups; however, the 6-month pad-weight leakage volumes are similar, and the percentage of subjects with >50% reduction in pad weight is identical at 69%. The cure rate, defined here as ≤1 g of leakage and per FDA guidelines,^24,25^ is also comparable between groups. Overall mean change from baseline showed a decrease of 73% for all subjects.

**Table 2.** Mean Pad-Weight Data at 1, 4, and 6-Month Follow-Up Visits

Group	Baseline pad weight	Subjects with a >50% reduction in pad weight from baseline			Cure rate (≤1 g leak)
		Mean pad-weight leakage volume (g)			
		1 month	4 months	6 months	6 months
All subjects (*n* = 29)	6.17 g	55.2%	75.9%	69.0%	65.5%
		2.14 g	1.31 g	1.69 g	
Group 1 (*n* = 16)	4.81 g	56.3%	68.8%	68.8%	68.8%
		2.19 g	1.19 g	1.81 g	
Group 2 (*n* = 13)	7.85 g	53.8%	84.6%	69.2%	61.5%
		2.08 g	1.46 g	1.54 g	

Percentage of subjects with a >50% reduction in pad weight (which represents a clinically meaningful decrease in leakage amount) and percentage of subjects considered cured at 6 months.

### Patient-reported outcomes

**FIG. 2.**
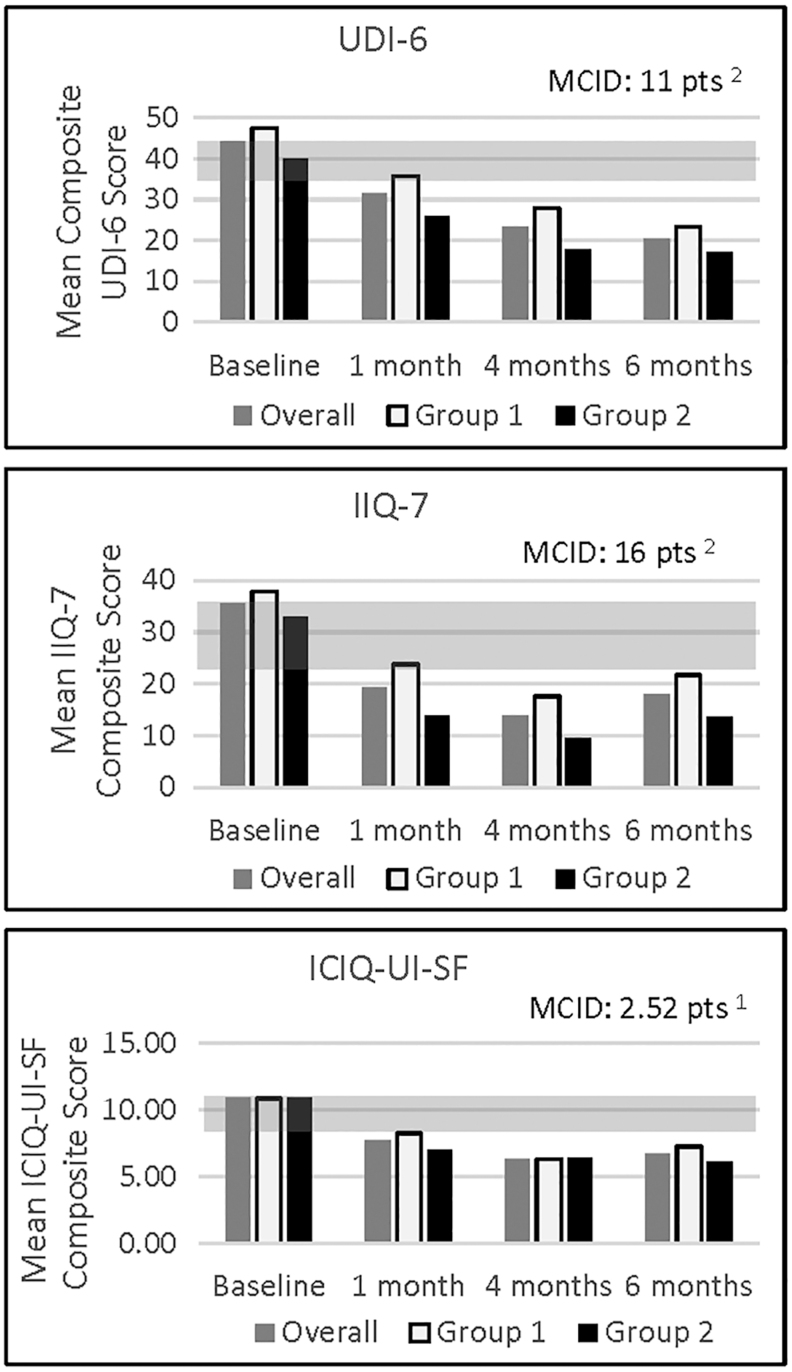
Mean composite scores for SUI-related patient-reported outcomes. The *gray bar* in each graph denotes a published MCID from the literature. ICIQ-UI-SF, International Consultation on Incontinence Modular Questionnaire-Urinary Incontinence-Short Form; IIQ-7, Incontinence Impact Questionnaire-Short Form; MCID, minimal clinical important difference; SUI, stress urinary incontinence; UDI-6, Urogenital Distress Inventory-6.

Clinically meaningful score decreases in subject's SUI symptoms, and improvement in quality of life was noted on two measures (UDI-6 and ICIQ-UI-SF) as early as 1-month post-treatment^22,23^ (Fig. 2). By 6-months post-treatment, all SUI-related subjective measures for both treatment groups show sustained improvement in mean composite scores. Group 1 reported greater mean composite scores than Group 2 at all measured time points for UDI-6 and IIQ-7.

### Seven-day bladder voiding diary

Subjects report a decrease in leakage episodes per day as soon as 1-month post-treatment (Table 3). By 6 months, ∼80% of subjects report less leakage episodes compared with baseline, with most reporting a ≥50% reduction from baseline. In addition, some subjects report an ability to resume strenuous physical exercise (*e.g.*, rock climbing) post-treatment.

**Table 3.** Mean Number of Daily Incontinence Episodes at 1, 4, and 6-Month Follow-Up Visits^*^

Group	Baseline mean # daily incontinence episodes	No. of incontinence episodes/day		
		% of subjects with >50% reduction from baseline in incontinence episodes/day		
		1 month	4 months	6 months
All subjects	2.0	1.2	1.2	1.0
(*n* = 29)		48%	69%	64%
Group 1	1.5	0.7	1.1	0.7
(*n* = 16)		50%	75%	60%
Group 2	2.7	1.7	1.3	1.3
(*n* = 13)		46%	62%	69%

^*^ *n* = 28 at 6-month time point.

### Safety

No unanticipated or serious adverse events have been reported in the trial to date. All other events were mild. One patient reported a urinary tract infection (UTI). The UTI occurred between the treatment visit and 1-month follow-up. The subject was treated with 1 week of antibiotics. The investigator assessed the UTI as unrelated to treatment.

## Discussion

This early feasibility study highlights the promising efficacy and safety of the Viveve System for the treatment of SUI. The results show continued benefit out to 6 months post-treatment, the time point assessed to date, for all subjects. Although the results are encouraging, caution should be exercised when interpreting the data since this is a small study and lacks a control group. A larger scale, randomized, blinded, sham-controlled study is warranted.

While the objective pad-weight data are similar between treatment groups, the subjective, SUI-related patient-reported outcome measures show slight differences in the mean composite scores, with Group 2 reporting decreased scores at almost all time points. This could be due to a lower baseline value for Group 2; or also because this was an unblinded study, therefore subjects knew whether they received one or two treatments. An analysis of 130 clinical trials showed a significant placebo effect in studies with continuous subjective outcomes, however little to no placebo effect for objective measures.^26^ In addition, the lack of a study-wide retention plan and unblinded nature of the study could account for the larger dropout rate from Group 1 (*n* = 5) versus Group 2 (*n* = 1). Interestingly, of the five women who dropped out of Group 1 before 6-month visit, all had improvement from baseline on the 1-hour PWT at their last measured visit; and two of the five had no leakage at all (0 g of leakage on the 1-hour PWT). Furthermore, based upon the Viveve System's proposed mechanism of action, which includes fibroblast activation and restoration of connective tissue of the lamina propria tissue layer,^27^ and what is known about collagen restoration, a second RF treatment done later (*e.g.*, 6 months vs. 6 weeks) may provide even greater benefit to women. A larger number of subjects, another study including a sham treatment group, a longer follow-up period, or a longer^28^ time between treatments may be necessary to determine the differences between one or two treatments and the optimal timing. Of note, this study will continue out to 12 months to assess safety and efficacy.

Although other RF devices are currently claiming to help treat SUI, no other studies with a nonablative, monopolar RF device have demonstrated a decrease in SUI symptoms as evaluated by objective measures (1-hour pad test and voiding diary) sustained out to 6-months post-treatment.^16,29–32^ This early feasibility study also includes validated SUI- and quality of life-related subjective questionnaires (UDI-6, IIQ-7, ICIQ-UI-SF) as study endpoints with >70% of women experiencing improvement (reduction from baseline) at 6 months. In addition, an earlier pilot study demonstrated the efficacy of the Viveve System for the treatment of SUI out to 12 months, with >90% response rate (reduction from baseline using validated SUI questionnaires). Although the IIQ-7 and ICIQ-UI-SF scores slightly increased from 4 to 6 months, the change was minimal and not clinically significant.^28,33^

These promising results were achieved with one treatment. Other RF devices require multiple (3+) sessions, typically at 1-month intervals.^34^ This incurs increased time and monetary cost to the patient, and increases the potential for a decrease in treatment compliance. It should also be noted that the Viveve System has a well-established safety profile. To date, thousands of women have been treated globally for sexual dysfunction with only mild adverse events reported.^15,35,36^ In the previously published, large-scale, randomized-controlled clinical trial using the Viveve System, the active and sham groups reported similar numbers of adverse events.^20^ In addition, recent ovine studies have confirmed tissue temperatures that would result in cellular responses related to the observed clinical outcomes with no thermal damage to the vaginal tissue following multiple pulses in the same area (Viveve internal data).

## Conclusions

While this article summarizes data from an early investigator-initiated feasibility study, the results include the first 6-month objective outcome data for the vaginal RF treatment for SUI. The Viveve treatment shows promise as a viable option for patients searching for minimally invasive nonsurgical treatments. Initial experience merits a larger scale, randomized, blinded, and sham-controlled study to investigate this treatment for SUI.

## Clinical Trial Registration

clinicaltrials.gov Identifier: NCT03066180.
